# Towards the Albino Mutant Gene in *Malus × Domestica* Borkh.

**DOI:** 10.3390/plants13233448

**Published:** 2024-12-09

**Authors:** Guodong Zhao, Yang Li, Linguang Jia, Dongmei Chen, Chaohong Zhang, Xinsheng Zhang, Fengqiu Yang, Tongsheng Zhao

**Affiliations:** Changli Institute of Pomology, Hebei Academy of Agricultural and Forestry Science, Qinhuangdao 066600, China; guodong19823@163.com (G.Z.); pxliyang01@163.com (Y.L.); dsjialinguang2020@163.com (L.J.); chendm2009@126.com (D.C.); 18931353275@163.com (F.Y.); tshzh71@163.com (T.Z.)

**Keywords:** *Malus domestica* Borkh., albino, mutant, shoot and leaf, mechanism

## Abstract

Albino mutation is among the most common phenomena that often causes a water imbalance and disturbs physiological functions in higher species of trees. Albinism frequently occurs in hybridized apples, but almost all seedlings die shortly after germination. In this study, a spontaneous albino mutant on Fuji apple trees was obtained. After bud grafting, new albino shoots with greenish-white leaves grew, although they were slender, small, and died easily. Resequencing analysis indicated that a total of 49.37 Gbp clean data of the albino mutant samples was obtained; its Q30 reached 91.43%, the average rate mapped was 93.69%, and genome coverage was 96.47% (at least one base cover). Comparisons of the sequences for the albino mutants revealed 4,817,412 single-nucleotide polymorphisms (SNPs), 721,688 insertion/deletion markers (InDels), and 43,072 structural variations (SVs). The genes with non-synonymous SNPs, InDels, and SVs in CDS were compared with KEGG, GO, COG, NR, and SwissProt databases, and a total of 5700 variant genes were identified. A total of 1377 mutant genes had the GO annotation information. Among these, 1520 mutant genes had the pathway annotation and took part in 123 pathways. A total of 1935 variant genes were functionally classified into 25 COG categories. Further research on these variants could help understand the molecular regulatory mechanism of the apple albino mutant. Similarly, variations in the homologous *MdAPG1* (Albino or pale-green mutant 1) gene, which was located on Chromosome 11 and belonged to the S-adenosyl-L-methionine-dependent methyltransferases superfamily, may have led to the generation of this apple albino mutant.

## 1. Introduction

Albinism is a common phenomenon in rice, soybean, wheat, tea trees, redwood, orange, and so on [1,2,3,4,5,6,7]. After bleaching of the leaf, the pigment content is rendered deficient, the density of stomata increases significantly, the chloroplasts in the guard cells and thin-walled cells are highly deficient, and the stomatal conductivity and respiration rate of the leaves are significantly increased at extremely high temperatures. Abnormal changes in the catheter tissue in albino branches disturb the water balance and physiological function in trees [1]. Apart from normal growth obstacles, albino plants are also vulnerable to pests and diseases and are often accompanied by lethal genes [8]. Hence, they were often considered meaningless mutations in the past.

Recently, the application value of albinism mutations has been redefined and has received increasing attention [4,7]. The contents of catechins in the leaves of the tea tree albino mutants ‘Baiye 1’ and ‘Huabai 1’ were significantly reduced, and the amino acid content was significantly increased. The tea made from the White Stem Tip was sweeter and more economical [9]. The *val1* mutant of rice is a narrow green albino leaf type, and its wild-type *VAL1* gene is a key enzyme gene that encodes purine biosynthesis, as well as influences the development of chlorophyll, chloroplast metabolic pathways, cell division during leaf growth, and several other growth and development processes [6]. The reduction in carbon nutrients results in chlorophyll loss, as well as affects some functions of the vascular system, such as water transportation, xylem synthesis, and development of the redwood albino mutant leaves [2].

Apple (*Malus domestica* Borkh.) is a well-known and widely cultivated fruit crop around the world. Numerous studies have been reported over the past few years [10,11]. In one study, 10 of 36 apple parents during the apple breeding program at the East Malling Research Station exhibited albino (gray-green) lethal hybrid types, while only 10 of the 500 seedlings derived from those parents had albino lethal genes [12]. Recently, a similar leaf disorder, zonal leaf chlorosis (ZLC), was observed on Honeycrisp, a widely grown apple cultivar in the US [13]. In another study, after a systematic analysis of 53 apple individuals, the progenies of 23 apple individuals showed heterozygous albino (gray-green) lethal phenotypes, including Golden Delicious, Jonathan, and Rome Beauty [14]. However, most studies report that the first young leaves of albino apple seedlings die, which poses difficulties for further research [8,15]. Obtaining and studying mutants is useful for understanding plant gene function, which helps us better understand the basic biological processes such as plant development [16], growth, metabolism [17], and stress responses [18,19]. In this study, a novel albino mutant was identified and analyzed based on naturally occurring mutations in the field. Based on the precision of high-throughput sequencing technology and the accuracy of bioinformatics analysis, acquired albino mutants were utilized to deeply explore genome-wide single-nucleotide polymorphisms (SNPs), insertion/deletion markers (InDels), structural variations (SVs), and copy number variations (CNVs) by comparing the genomic differences between mutants and wild types. This allows us to fully understand the site variation information of albino mutations, thereby providing a basis for accurately locating and verifying the gene mutations that lead to the albino phenotype. It has potential application value for plant development and physiological research.

## 2. Results

### 2.1. Identification of Albino Mutations in Apple

The white mutant in the apple orchard was the branch of the Fuji apple 15 years after planting. The albino branches were greenish-white, slender, and short (no more than 20 cm). After removal, new white shoots grew again from the base of that Fuji apple tree. The leaves of these branches were also greenish-white, much thinner, and smaller than the normal ones. 

**Figure 1:**
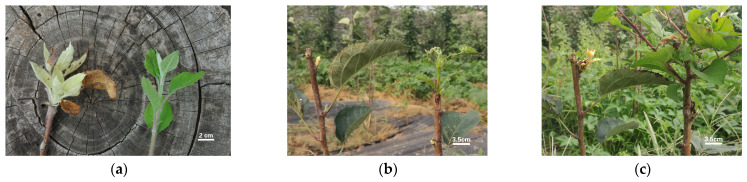
Phenotypic observations of albino mutant and its wild type in the apple orchard: (**a**) The observed phenotype of the mutant and wild type, which was collected from the individual mother plant. (**b**) The expression of albino mutants that were grafted onto SH6 interstock for 30 days. (**c**) The expression of albino mutants that were grafted onto SH6 interstock for 60 days.

To compare the growth properties of green and albino shoots, we collected both and grafted them on SH6 interstock. Six buds on the albino shoots were grafted onto SH6 interstock, of which two survived after about two months of growth. The albino shoots on the SH6 interstock showed similar behavior during the first year of culture. New shoots with white leaves appeared slowly, whereas the control bud quickly grew green and thick shoots. After two months, the new albino branches on the SH6 interstock still measured <10 cm and died during the winter (Figure 1).

### 2.2. Resequencing Analysis of the Albino Mutants

Based on the filtered sequenced reads, 164,577,988 and 164,928,294 clean reads of the albino mutant and the corresponding control were obtained, of which more than 91.12% and 91.73% had Q scores at Q30 level (Table 1), indicating high-quality reads worthy of further analysis. All the clean reads were mapped onto the apple reference genome in the NCBI for gene annotation. More than 92% (92.23% and 95.15%) of reads of each sample were mapped with the apple genome, and the ratio of dual–terminal sequencing sequences was 81.36% and 75.78%, respectively. The average sample GC content was 38.84%, and the distribution of GC of each sample was normal.

### 2.3. Detection and Annotation of SNPs 

**Figure 2:**
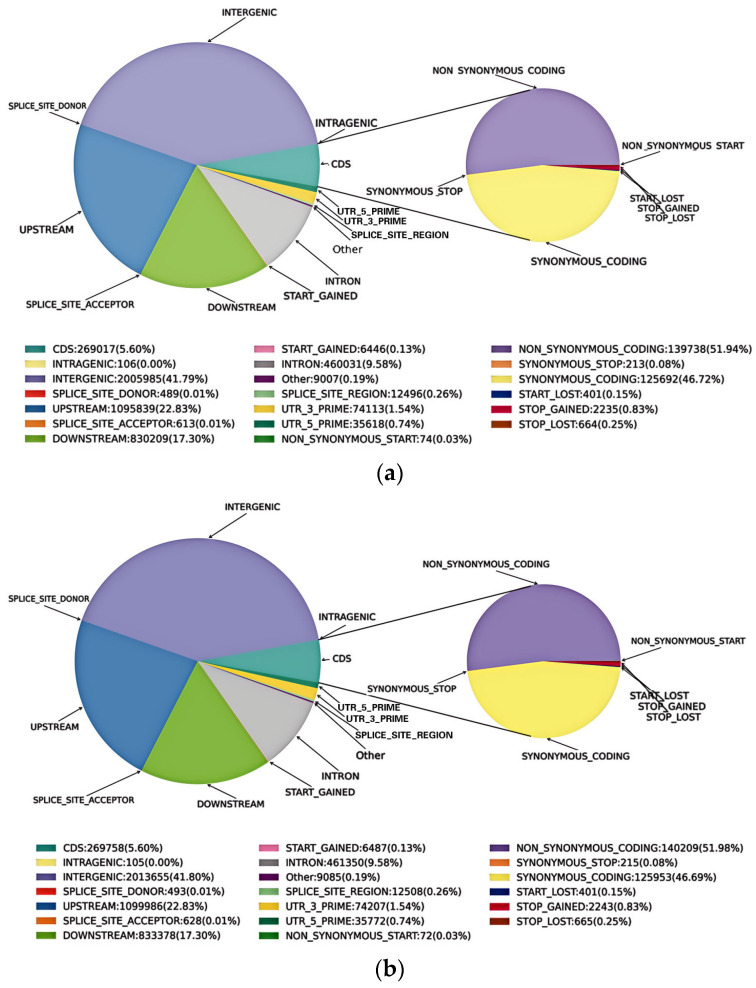
Annotation of the detected SNPs in the apple albino mutant (**a**) and its wild type (**b**).

The number of SNPs was detected for each sample (Table 2). A total of 4,817,412 SNPs were detected in the apple albino mutant, which comprised 68.36% transitions and 31.64% transversions, and 71.70% were heterozygous (Table 2). A total of 4,799,969 SNPs were detected in the wild type, which comprised 68.35% transitions and 31.65% transversions, and 71.62% were heterozygous. In addition, transition–transversion ratios (Ti/Tv) for all heterozygous and homozygous SNPs were obtained, with an average of 2.15 for all samples (Table 2). The annotation results of SNPs indicate that most SNPs were located in intergenic regions (41.80%), and 5.60% of SNPs were located in coding sequence (CDS) regions (Figure 2). In CDS, 140,209 non-synonymous ones (amino acids in the coding region changed) and 125,953 synonymous ones (amino acids in the coding region are not changed) were observed for the apple albino mutant, and 139,738 non-synonymous and 125,692 synonymous ones were observed for the wild type. 

### 2.4. Detection and Annotation of InDels

**Figure 3:**
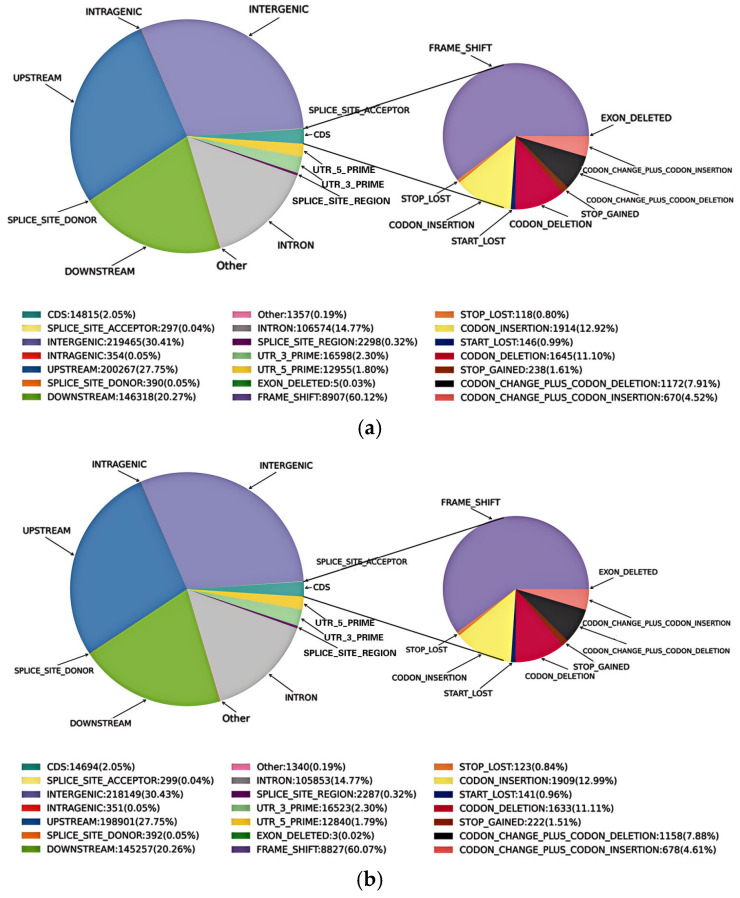
Annotation of the detected InDels in the apple albino mutant (**a**) and its wild type (**b**).

Furthermore, a total of 721,688, 14,815 InDels were identified in genome and CDs for the albino mutant (Table 3); among the detected InDels in CDs, there were 6893 insertions and 7922 deletions, and 73.49% were heterozygous. For the wild type, 716,886 and 14,694 InDels were identified in the genome and CDs, respectively. Among these detected InDels in CDs, there were 6850 insertions and 7844 deletions, and 73.16% were heterozygous. Annotation results indicate that most InDels were frameshift mutations (60.07–60.12%) (Figure 3).

### 2.5. Detection and Annotation of the SVs

A total of 43,072 SVs were detected for the albino mutant (Table 4), which comprised 33.11% insertions (INS), 52.28% deletions (DEL), 1.90% inversions (INV), 6.25% intra-chromosomal translocations (ITX), and 6.05% inter-chromosomal translocations (CTX). A total of 43,823 SVs were detected for the wild type, which comprised 32.83% INS, 56.80% DEL, 1.72%INV, 5.69% ITX, 2.83% CTX. According to the annotation result of SVs for each sample, SVs mainly occur in intergenic regions (Table 5).

### 2.6. Identification and Functional Annotation of the DNA-Level Variant Genes 

Mutations that occur in CDS regions may cause changes in gene function [20]. The detected genes of non-synonymous SNP polymorphism, InDel mutations, and SV mutations in the coding area are presented in Table 6. The albino mutant and its mother plant showed 48,345 and 48,532 variant genes, respectively, including SNP mutation genes (32,039 and 32,003), InDel mutation genes (10,542 and 10,481), and SV mutation genes (5764 and 6098). Functional annotation was conducted by comparing the genes against functional databases using the sequence alignment tool BLAST, in which 5700 variant genes received the annotated information for albino mutants.

**Figure 4:**
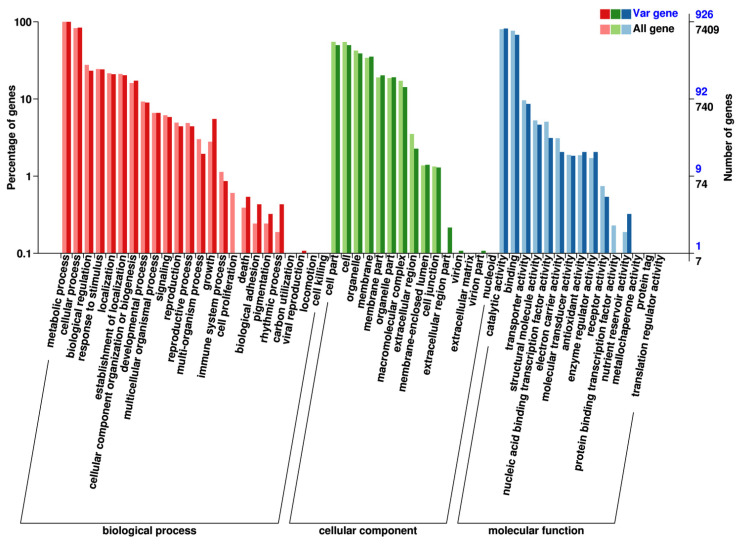
GO classification of the variant genes of apple albino mutant.

A total of 1377 variant genes were annotated with GO annotation analysis. These variant genes were classified into three main categories, biological process, cellular component, and molecular function, which could be further classified into 54 sub-categories (Figure 4). Significant differences between the distributions of GO terms for the variant genes compared with the whole genome were used to reveal the functional significance of the changes observed. Among the biological processes, cell growth, cellulose microfibril organization, and Golgi vesicle transport were enriched in the variant genes compared with the whole genome. Among the cellular components, the anchored component of the membrane and endoplasmic reticulum membrane were enriched. Among the molecular functions, protein kinase activity was enriched (Table 7). 

**Figure 5:**
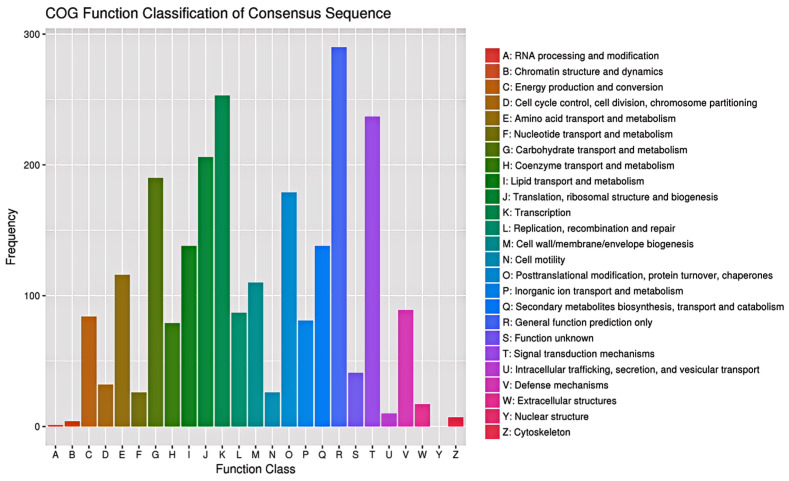
COG classification of the variant genes of apple albino mutant.

For COG classification, 1935 variant genes were functionally classified into 25 COG categories (Figure 5). The COG clusters revealed that general function prediction (290, 15%), transcription (253, 13%), and signal transduction mechanisms (237, 12%) constituted the major groups of variant genes. Furthermore, among the 92 annotated variant genes linked to chloroplasts, 13 variant genes were involved in the biosynthesis, transport, and catabolism of secondary metabolites, 11 were involved in posttranslational modification, protein turnover, and chaperones, 8 were involved in energy production and conversion, 7 were involved in carbohydrate transport and metabolism, and 7 were involved in amino acid transport and metabolism.

**Figure 6:**
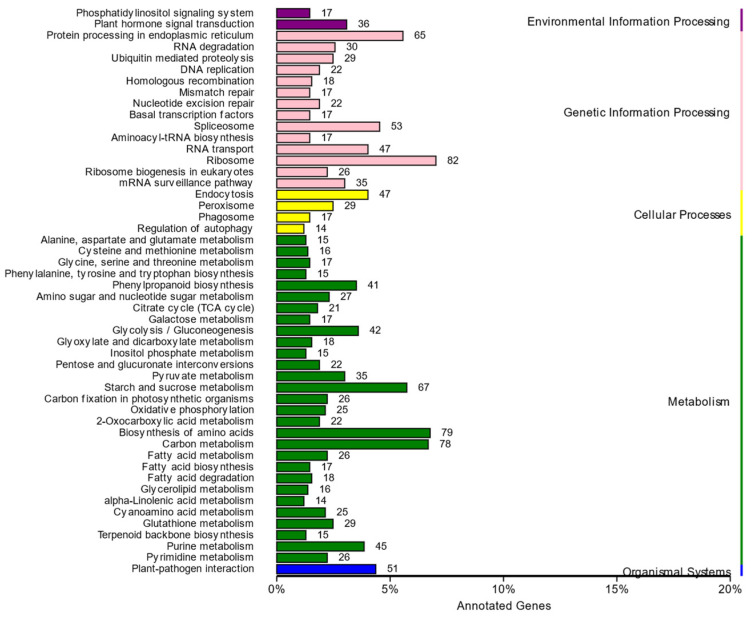
KEGG function classification for the variant genes of apple albino mutant.

**Figure 7:**
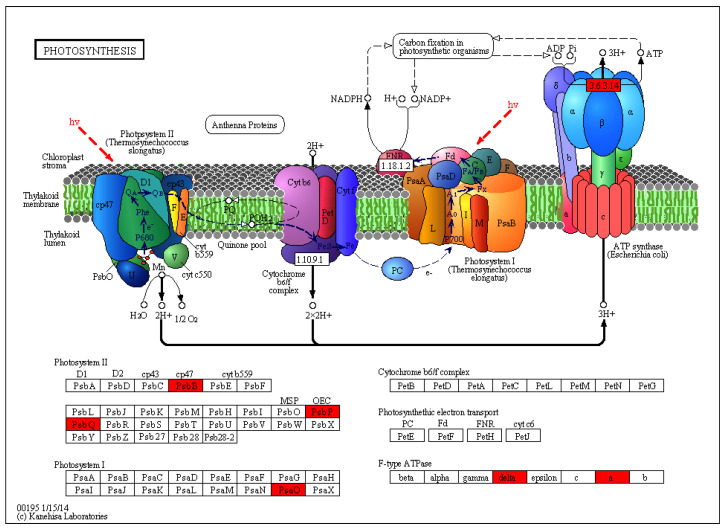
Photosynthesis pathway of the apple albino mutant. The characters in the box represent the enzyme encoded by the corresponding gene. The whole pathway includes many different enzymes formed by complex biochemical reactions, and the variation genes associated with this pathway are marked with red boxes. The image was retrieved from KEGG (www.kegg.jp/pathway/mdm00195 (accessed on 15 June 2024)) and adapted for reference.

A total of 1520 variant genes were annotated after a search against the KEGG database. The metabolic pathways of these variant genes were classified into 123 pathways, with the ribosome, biosynthesis of amino acids, and carbon metabolism pathways accounting for a large proportion of the variant genes (Figure 6). Furthermore, seven variant genes were identified in the photosynthesis pathway that were mainly involved in the biosynthesis of several photosystem II proteins and the synthesis of the partial subunit of photosystem-I and F-type ATPase (Figure 7).

### 2.7. Blast Alignment for Variant Genes for CDS Sequences

For this study, the CDS sequence of four *Arabidopsis* albinism genes was collected in GenBank (https://www.ncbi.nlm.nih.gov/), (accessed on 7 November 2023), containing *APG1* (accession numbers: NM_116206.3), *APG2* (accession numbers: NM_126173.4), *APG10* (accession numbers: NM_129181.4), *ALB3* (accession numbers: NM_001124933.1) [21,22,23,24]. These sequences were compared with the sequenced apple albino mutant using BLAST. Only NM_116206.3 was retrieved in the apple gene *MD11G1277500* (physical position: 39474405-39476743), suggesting that the albino variety of the ‘Fuji’ apple tree here might be related to genetic variations in chromosome 11, and variations in the homologous *MdAPG1* gene led to the generation of the apple albino mutant. This gene belongs to the S-adenosyl-L-methionine-dependent methyltransferases superfamily, encodes an MPBQ/MSBQ methyltransferase that is located in the chloroplast inner envelope membrane and is involved in the biosynthesis of plastoquinone (PQ) [25]. Mutants lacking PQ cannot survive beyond the seedling stage and contain decreased numbers of lamellae with reduced levels of chlorophyll [23,26]. Similarly, we found 11 SNPs from chromosome 11 (Table 8), which showed some specific nucleotide insertion events leading to the alteration, and we hope that *MdAPG1*-associated SNPs would be helpful for accelerating the development of albino markers.

## 3. Discussion

When plants are affected by ion (spatial) radiation, drug induction, and artificially controlled hybridization during the growth process, a few albino tissues or plants often appear [27]. Albino lethal mutations of soybeans were obtained by the introduction of exogenous DNA from pollen tube channels into soybeans [28]. The mutation of ethyl methyl sulfonic acid (EMS)-treated *Arabidopsis* resulted in several albino mutants [29]. The callus of shellfish was cultured under a 12 h light/dark cycle, and the albino stem segment was induced [27]. In the M2 group of spatial mutagenesis-induced light-bodied rice cultivar Francis, a green, multivariate tiller dwarf mutant was found [30]. The discovery of several albino mutations, including the spontaneous albino mutations in Fuji apple in the present study, provides a good experimental basis for the study of the physiological functions, genetic regulation, and related gene excavation of albino tissues. It also helps reveal and understand the functions of key genes in complex biological processes, such as chloroplast development.

Several studies have reported that albino plants or tissues are mostly caused by abnormal metabolic processes in pigment cells, tissues, and even organs, especially those associated with photosynthesis. Chlorophyll (a) and (b) were not present in the albino variants of *Arabidopsis* [29]. The leaf vein white mutant of Jinhui No. 10 (*Oryza sativa* L.) showed normal performance during the seedling stage, with whitening in the primary and secondary midrib of the first, second, and third leaves during the late booting stage, whereas the mesophyll cells showed no significant change [31]. Compared with the wild type, the content of chlorophyll (a) and carotenoids in Ningjing 36 albino mutants decreased significantly, the number of normal chloroplasts decreased significantly and contained several small abnormal chloroplasts, and there was no fully developed cystic layer structure in the abnormal chloroplasts [32]. When the growth and developmental conditions are changed, some albino tissues can be restored, for example, when a part of the albino tissue was treated with a photoperiodic incubation, it changed its original light and dark cycle [27]. For albino mutant *tsa1* in rice, bleaching of the blade was observed at 20–24 °C (low temperature), while there was no significant difference between the albino and wild-type at 28–32 °C (higher temperature) [32]. However, to date, no studies have yet been performed on the restorability of the current albino phenotype into the original wild type.

Albinism is controlled by genetics or epigenetics [33], and controlling albinism is extremely complex. Previous studies have demonstrated that genes of various albino mutations were located on chromosomes, such as Chr2, Chr4, Chr5, Chr6, and Chr8 in rice [30,31,32,34,35,36]. Reports on albinism in apples began in the 1930s when researchers discovered the emergence of albino plants in apple hybrids, with a maximum albinism rate of 22.3% [8]. Subsequent studies reported that the albino genes in individuals or cultivars, such as the ‘Golden Crown’, were close to the genetic distance of the resistance gene *V_f_* of the Apple Black Star disease [12]. Moreover, the pale-green lethal (PGL) gene was mapped to the top of the Linkage Group 16, and its associated lethality was expressed only in homozygous recessive *pgl1pgl1* genotypes. Furthermore, the LG9-H1 haplotype from the offspring of ‘Honeycrisp’ indicates that this genomic region is a good target for further studies into the genetics and physiology of *ZLC* [13]. Besides, one new albino mutant, belonging to the S-adenosyl-L-methionine-dependent methyltransferases superfamily located in chromosome 11 of apple in this experiment, might lay a foundation for further exploration and utilization of apple albino genes. In this study, we search for mutations that occurred in coding DNA sequence; however, gene silencing or reduced expression might also be caused by a polymorphism within an intron or even promotor region, and further analysis would be useful to discover unknown variants in the apple albino mutant. 

The traditional genetic approach remains one of the most successful strategies for mutated gene screening in plants [37]. However, pinpointing potentially interesting candidate genes depends on maximizing the amount of recombination (genetic mapping). In this study, mutant shoots remained small in size, usually died within a few months, and never showed flower development. Next-generation sequencing (NGS) technology, such as whole-genome resequencing (WGR), can identify the mutation sites directly by sequencing the entire genome of the mutant and comparing it with the wild-type genome [38]. Although such localization results may not explicitly indicate which candidate genes correspond to the mutant phenotype, complementary subsequent experiments such as fluorescence-based quantitation assays could be used for further gene functional confirmation.

## 4. Materials and Methods

### 4.1. Plant Materials

In June 2017, a natural white mutant (albino) Fuji apple plant was identified in an orchard located in Xinglong County, Hebei Province, China, which employed the SH6 interstock for high-density cultivation. In June 2018, the mutants and normally growing branches were collected from the mother plant for further study.

### 4.2. Identification of Albino Mutants

The experimental materials were grafted onto SH6 interstock to minimize the influence of the rootstock on their growth and development. Initially, we established the base rootstock and grafted the SH6 interstock 10 cm above ground level. During the growth period of the SH6 interstock, we performed pruning to retain two branches for subsequent cultivation. In mid to late June, we grafted both the mutants and their mother plants onto the branches of the SH6 interstock. The mutant and wild-type phenotypes reappeared on the respective sprouting budwood when the sprouts grew into branches of about 10 cm. 

### 4.3. Resequencing Analysis of Albino Mutants

Leaves of apple albino mutants and their mother plants were collected, and DNA was extracted using CTAB. Using the Illumina HiSeq 4000 PE125 sequencing strategy, the paired-end sequencing approach was used to construct and sequence the DNA small fragment library (building a 500 bp DNA library), as well as obtain sequencing data. Then, original reads were quality assessed and filtered to generate clean reads for subsequent alignment analysis: (1) remove reads with adapters; (2) remove sequences with N base ratios greater than 10% in reads; and (3) remove reads if more than 50% of the bases had a base quality value Q < 10 in sequenced fragments. To estimate the sequencing quality after clean data were determined, Q scores were calculated using the formula Q = −10log_10_(e), where e means sequencing error rate. A quality of Q30 means that the base call accuracy is 99.9%. 

### 4.4. Detection and Annotation of SNPs, INDELs, and SVs 

The clean reads from each sample were aligned to the apple GDDH13 reference genome (v1.0) using the Burrows–Wheeler Aligner software (BWA, 0.7.17-r1188) [39], Samtools was used for mark duplicates, and then GATK was used for local realignment and base recalibration [40]. An SNP set was formed by combining GATK and Samtools SNP calling analysis with default parameters. Structural variations (SVs) were detected using BreakDancer, containing large fragments of SV types, including insertions (INS), inversions (INV), deletions (DEL), intra-chromosomal translocations (ITX), and inter-chromosomal translocations (CTX). SNPs, INDELs, and SVs were annotated using snpEff. The localizations of the SNPs, InDels, and SVs were based on the annotation of reference genome databases.

### 4.5. Explanation of Albino Gene in Apple

The non-synonymous SNP polymorphism mutations, InDell mutations, and SV mutations were detected in the coding area. The DNA-level variant genes were annotated with KEGG, GO, COG, NR, and SwissProt databases. Simultaneously, the CDS sequence of plant albinism genes was searched on GenBank and compared with the sequenced albino plants.

## Figures and Tables

**Table 1:** Analysis of sequencing data of the apple albino mutant and its wild type.

Samples	% ≥ Q30	Total Reads	Mapped Reads (%)	GC Content	Cov Ratio 1× (%)	Cov Ratio 5× (%)	Cov Ratio 10× (%)
Albino mutant	91.12	164,577,988	95.15	38.73	96.51	94.38	92
Wild type	91.73	164,928,294	92.23	38.95	96.42	94.14	91.28

**Table 2:** Genetic variation in SNPs detected in the apple albino mutant and its wild type.

Samples	SNP Number	Transition	Transversion	Ti/Tv	Heterozygosity	Homozygosity	Het-Ratio
Albino mutant	4,817,412	3,293,167	1,524,245	2.16	3,454,111	1,363,301	71.70%
Wild type	4,799,969	3,280,841	1,519,128	2.15	3,437,848	1,362,121	71.62%

**Table 3:** Genetic variation in InDels detected in the apple albino mutant and its wild type.

Samples	CDS-Insertion	CDS-Deletion	CDS-Homo	CDS-Het	CDS-Total	Genome-Insertion	Genome-Deletion	Genome-Homo	Genome-Het	Genome-Total
Albino mutant	6893	7922	3077	11,738	14,815	342,497	379,191	191,289	530,399	721,688
Wild type	6850	7844	3094	11,600	14,694	339,728	377,158	192,379	524,507	716,886
Total	7371	8259	--	--	15,630	358,480	391,197	--	--	749,677

**Table 4:** Genetic variation in SVs detected in the apple albino mutant and its wild type.

Samples	SV	INS	DEL	INV	ITX	CTX	UN
Albino mutant	43,072	14,261	22,520	855	2692	2608	136
Wild type	43,823	143,88	24,892	753	2492	1239	59

**Table 5:** Annotation of the detected SVs in the apple albino mutant and its wild type.

Samples	Type	Exon	Intron	Intergenic
Albino mutant	DEL	3149	3152	16,219
	INS	2942	2324	8995
	INV	471	59	325
	Total	6562	5535	25,539
Wild type	DEL	3541	3526	17,825
	INS	2895	2256	9237
	INV	405	57	291
	Total	6841	5839	27,353

**Table 6:** Summary of genetic variations in the apple albino mutant and its wild type.

Samples	Genes with Non-Synonymous SNP	Genes with InDel	Genes with SV	Total
Albino mutant	32,039	10,542	5764	48,345
Wild type	32,003	10,481	6098	48,582

**Table 7:** GO enrichment analysis of the variant genes of apple albino mutant.

GO ID	GO Term	Variant Genes Number	Corrected *p*-Value
GO:0016049	cell growth	40	8.21 × 10^−11^
GO:0010215	cellulose microfibril organization	34	7.06 × 10^−9^
GO:0048193	Golgi vesicle transport	13	0.0423
GO:0031225	anchored component of membrane	34	1.42 × 10^−8^
GO:0005789	endoplasmic reticulum membrane	14	0.0100
GO:0004672	protein kinase activity	37	6.95 × 10^−5^

**Table 8:** SNP and InDel variations in *MdAPG1* gene.

Chromosome	Position	Reference	Alteration	Mutation Type	SNPEFF_EFFECT
Chr11	39,476,519	T	TC	Insertion	UTR_3_PRIME; SNPEFF_TRANSCRIPT_ID=mRNA:MD11G1277500
Chr11	39,475,586	A	AT	Insertion	INTRON; SNPEFF_TRANSCRIPT_ID=mRNA:MD11G1277500
Chr11	39,473,770	G	GT	Insertion	UPSTREAM; SNPEFF_TRANSCRIPT_ID=mRNA:MD11G1277500
Chr11	39,473,102	C	CT;CTCTT	Insertion	UPSTREAM; SNPEFF_TRANSCRIPT_ID=mRNA:MD11G1277500
Chr11	39,474,458	T	TTCTCTCTCTC	Insertion	UTR_5_PRIME; SNPEFF_TRANSCRIPT_ID=mRNA:MD11G1277500
Chr11	39,476,666	CAATTTCATTTGGG	C	Insertion	UTR_3_PRIME; SNPEFF_TRANSCRIPT_ID=mRNA:MD11G1277500
Chr11	39,469,526	GA	G	Deletion	UPSTREAM; SNPEFF_TRANSCRIPT_ID=mRNA:MD11G1277500
Chr11	39,474,267	CAG	C	Deletion	UPSTREAM; SNPEFF_TRANSCRIPT_ID=mRNA:MD11G1277500
Chr11	39,474,636	TATCAA	T	Deletion	UTR_5_PRIME; SNPEFF_TRANSCRIPT_ID=mRNA:MD11G1277500
Chr11	39,476,499	T	TGA	Insertion	FRAME_SHIFT; SNPEFF_TRANSCRIPT_ID=mRNA:MD11G1277500
Chr11	39,474,094	CCA	C	Deletion	UPSTREAM; SNPEFF_TRANSCRIPT_ID=mRNA:MD11G1277500

## Data Availability

The datasets generated during the current study are available from the corresponding author upon reasonable request.
